# Preoperative prediction of tumor deposits in rectal cancer with clinical-magnetic resonance deep learning-based radiomic models

**DOI:** 10.3389/fonc.2023.1078863

**Published:** 2023-02-20

**Authors:** Chunlong Fu, Tingting Shao, Min Hou, Jiali Qu, Ping Li, Zebin Yang, Kangfei Shan, Meikang Wu, Weida Li, Xuan Wang, Jingfeng Zhang, Fanghong Luo, Long Zhou, Jihong Sun, Fenhua Zhao

**Affiliations:** ^1^ Department of Radiology, Affiliated Dongyang Hospital of Wenzhou Medical University, Dongyang, China; ^2^ Department of Radiology, Sir Run Run Shaw Hospital, Zhejiang University School of Medicine, Hangzhou, China; ^3^ Department of Radiology, Jiaxing Hospital of Traditional Chinese Medicine, Jiaxing, China; ^4^ Key Laboratory of Diagnosis and Treatment of Digestive System Tumors of Zhejiang Province, Ningbo, China; ^5^ Cancer Center, Zhejiang University, Hangzhou, China

**Keywords:** deep learning, rectal cancer, tumor deposit, magnetic resonance imaging, diffusion-weighted imaging

## Abstract

**Background:**

This study aimed to establish an effective model for preoperative prediction of tumor deposits (TDs) in patients with rectal cancer (RC).

**Methods:**

In 500 patients, radiomic features were extracted from magnetic resonance imaging (MRI) using modalities such as high-resolution T2-weighted (HRT2) imaging and diffusion-weighted imaging (DWI). Machine learning (ML)-based and deep learning (DL)-based radiomic models were developed and integrated with clinical characteristics for TD prediction. The performance of the models was assessed using the area under the curve (AUC) over five-fold cross-validation.

**Results:**

A total of 564 radiomic features that quantified the intensity, shape, orientation, and texture of the tumor were extracted for each patient. The HRT2-ML, DWI-ML, Merged-ML, HRT2-DL, DWI-DL, and Merged-DL models demonstrated AUCs of 0.62 ± 0.02, 0.64 ± 0.08, 0.69 ± 0.04, 0.57 ± 0.06, 0.68 ± 0.03, and 0.59 ± 0.04, respectively. The clinical-ML, clinical-HRT2-ML, clinical-DWI-ML, clinical-Merged-ML, clinical-DL, clinical-HRT2-DL, clinical-DWI-DL, and clinical-Merged-DL models demonstrated AUCs of 0.81 ± 0.06, 0.79 ± 0.02, 0.81 ± 0.02, 0.83 ± 0.01, 0.81 ± 0.04, 0.83 ± 0.04, 0.90 ± 0.04, and 0.83 ± 0.05, respectively. The clinical-DWI-DL model achieved the best predictive performance (accuracy 0.84 ± 0.05, sensitivity 0.94 ± 0. 13, specificity 0.79 ± 0.04).

**Conclusions:**

A comprehensive model combining MRI radiomic features and clinical characteristics achieved promising performance in TD prediction for RC patients. This approach has the potential to assist clinicians in preoperative stage evaluation and personalized treatment of RC patients.

## 1 Introduction

Colorectal cancer (CRC) is the third most common malignancy and second leading cause of death worldwide. In particular, rectal cancer (RC) accounts for approximately one-third of CRC (1). A tumor deposit (TD) is defined as a discontinuous cancerous nodule located in the mesenteric fascia without obvious nodal or vascular features. The median incidence of TDs in CRC patients is approximately 21.3% (2). Positive TDs can elevate clinical stages of RC patients. RC patients with positive TDs are classified as N1c and treated as clinical stage III, in the absence of nodal metastases. The efficacy of adjuvant chemotherapy in stage III colon cancer had been widely recognized in previous studies, For the TD patients, with the receipt of chemotherapy had decreased risk of cancer-specific mortality compared with those not (3). However, only 52% of TD-positive and lymph node (LN)-negative patients receive preoperative adjuvant chemotherapy (4). Therefore, early identification of TDs is important and valuable for stage evaluation and treatment planning.

Magnetic resonance imaging (MRI) is considered the most reliable imaging modality for the initial pretreatment evaluation of patients with RC, including the assessment of TN staging, circumferential resection margin (CRM), and extramural vascular invasion (EMVI). Moreover, it can assist in the clinical preoperative management of RC patients, determination of surgical scope, and assessment of treatment response to neoadjuvant therapy (5, 6). However, MRI is limited in determining tumor spread in the mesorectum. Gröne et al. used a small diameter of 5 mm as the statistical threshold to determine N staging. With this value, the sensitivity, specificity, and accuracy of MRI staging were 72%, 45.7%, and 56.7%, respectively (7). Langman et al. showed that mesenteric nodules <3 mm had a 28% probability of malignancy (8). These studies focused on the presence of tumor spread in mesorectal nodules, either LN metastasis or TDs. However, the evaluation of malignant LNs alone is insufficient to reflect the actual spread of RC in the mesentery. According to the current European Society for Medical Oncology (ESMO) preoperative risk assessment criteria, patients with TDs are classified into a high-risk group with a worse prognosis (9). A previous study confirmed that the presence of TDs is an independent risk factor for the prognosis of patients with RC (10). An analysis of two prognostic studies in N0 and N1c stages showed a significant difference in the five-year survival rates (N0, 91.5%; N1c, 37%) (11). Therefore, preoperative determination of tumor deposition status in patients with RC is essential for optimal treatment.

Currently, the presence of TDs is determined by pathological analysis after radical tumor resection. However, this method is invasive and can be performed only postoperatively. According to a previous study, MRI can help preoperatively and identify TDs and LN metastases, as these lesions appear to have distinguishable imaging characteristics on MRI (12). However, TDs are usually less than 5 mm in diameter, and identifying such small nodules and accurately assess the characteristics of the nodule can be challenging for radiologists who are already overburdened in reading MRI in daily practice.

By extracting vast amounts of quantitative features from imaging and providing non-visual information that indicates the biological behavior of tumors, radiomics has gained popularity for the non-invasive prediction of clinical or prognostic features of tumors, such as T staging of RCs, LN status, vascular and nerve invasion, distant metastasis, and pathological complete response to neoadjuvant chemotherapy (13–20). Meanwhile, artificial intelligence including deep neural networks has demonstrated high performance in the analysis of medical images (21–23), providing cancer risk assessment, recurrence, and survival predictions with higher accuracy than human experts. Recently, several radiomic models have been developed based on ultrasound (US), computed tomography (CT), and MRI to preoperatively predict TDs in patients with RC (10, 24, 25). However, the sample sizes in these studies were relatively small. Furthermore, the MRI study carried out by Yang et al. (25) only extracted the radiomic features from high-resolution T2 weighted (HRT2) MRI, whereas functional MRI, such as diffusion-weighted imaging (DWI), carries more information on the heterogeneity of tumors. Currently, there is a lack of functional MRI-based deep-learning (DL) radiomics research in this field.

This study aimed to develop an MR-based DL radiomic model for preoperative TD prediction in a larger cohort with higher prediction accuracy. This model extracts radiomic features from both HRT2 and DWI images and integrates clinical factors into TD prediction.

## 2 Methods

### 2.1 Patient characteristics

**Figure 1:**
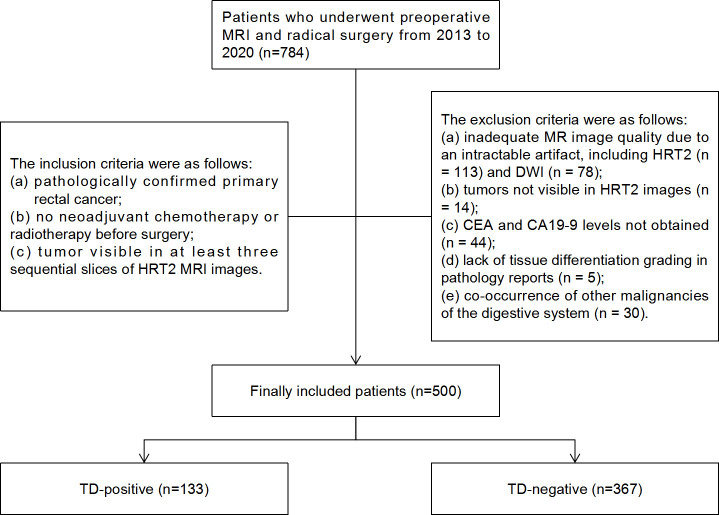
Flowchart of patient selection and TD distribution in the study. TD, tumor deposition; CEA, carcinoembryonic antigen; CA19-9, carbohydrate antigen 19-9.

The records of 784 consecutive RC patients who underwent preoperative MRI and radical surgery between 2013 and 2020 at Sir Run Run Shaw Hospital affiliated with Zhejiang University School of Medicine were reviewed retrospectively. The local institutional review board approved this study and provided a waiver of consent. The inclusion criteria were: (a) pathologically confirmed primary RC; (b) no neoadjuvant chemotherapy or radiotherapy before surgery; and (c) tumor visible in at least three sequential slices of HRT2 MRI. The exclusion criteria were as follows: (a) inadequate MRI quality due to intractable artifacts, including HRT2 (n = 113) and DWI (n = 78); (b) tumors not visible in HRT2 images (n = 14); (c) carcinoembryonic antigen (CEA) and carbohydrate antigen 19-9 (CA19-9) levels not obtained (n = 44); (d) lack of tissue differentiation grading in pathology reports (n = 5); and (e) co-occurrence of other digestive system malignancies (n = 30). Ultimately, 500 patients were enrolled in this study (**Figure 1**).

### 2.2 Clinical characteristics and pathological criteria

Clinical characteristics, including sex, age, body mass index (BMI), and carbohydrate antigen 19-9 (CA19-9) and carcinoembryonic antigen (CEA) levels, were collected from electronic medical records. Histological grading, pathological tumor node metastasis (pTNM) staging, LNs, TDs, vascular and nerve invasion, and other clinical data were obtained from pathological reports. The eighth edition of the AJCC staging system was used as a reference for the pTNM staging. TDs are defined as discrete tumor foci in the pericolic or perirectal fat, without histological evidence of residual lymph node or identifiable vascular or neural structures.The distance between the tumor and anus, TN staging, CRM, and EMVI based on the MRI were obtained from standardized reports of the picture archiving and communication system (PACS). The criteria for determining LN positivity on MRI were based on the latest recommendations of the 2016 European Society of Gastrointestinal and Abdominal Radiology consensus meeting (26). MRI tumor length was defined as the T2 sagittal tumor length. The distance from the anus was defined as the distance from the most inferior boundary of the tumor to the subcutaneous edge of the anus. The criterion for EMVI positivity was tumor invasion of the extramural vessels, with or without vessel dilatation (27). CRM positivity was defined as a tumor location within 1 mm of the mesorectal fascia, including suspicious LNs, TDs, tumor expansion, and EMVI (28). Unclear or missing information in the MRI reports were labeled and finally confirmed by a radiologist with nine years of working experience.

### 2.3 MRI scanning

MRI acquisitions were performed using the following 3.0-T MRI scanners: Signa HDxt (GE Healthcare, Chicago, IL), Discovery MR750w (GE Healthcare), and MAGNETOM Skyra (Siemens Healthineers, Erlangen, Germany). The MRI protocol consisted of one axial HRT2 MRI sequence and one DWI acquisition obtained using b-values of 0 and 1,000 (or 800) s/mm². No intravenous contrast agents were administered. Details of the MRI acquisition parameters are listed in **Table 1**.

**Table 1:** Image acquisition parameters.

Machine type	GE-Signa HDxt		GE-Discovery MR750W		SIEMENS-Skyra	
Modality	HRT2	DWI	HRT2	DWI	HRT2	DWI
**Repetition time (ms)**	3300	5900	3300	8000	5800	4400
**Echo time (ms)**	130	66	120	66	99	61
**Slice thickness (mm)**	3	5	3	5	3	5
**Slice gap (mm)**	0.3	1	0.3	1	0.3	1
**Matrix**	512×512	256×256	512×512	256×256	320×410	128×160
**Echo train length**	20	1	20	1	18	1
**FOV (mm**×**mm)**	160×160	250×250	160×160	380×380	160×160	300×300
**b-values (s/mm2)**		800		1000		800

### 2.4 Tumor segmentation and processing

Before image segmentation, patient-sensitive information was anonymized. The primary tumor region (3D volume) was semi-manually segmented on axial HRT2 and DWI images by a junior radiologist (with more than three years of experience in radiology) using an open-source software tool (ITK-SNAP 3.8; www.itksnap.org) (29). Automatic tumor segmentation using a CE-net-based DL segmentation model (30) was performed on the axial HRT2 images to assist radiologists. All segmentation masks were reviewed by a senior radiologist (with more than five years of experience in radiology) and finally confirmed by another senior radiologist (with more than 10 years of experience in radiology). Disagreements were resolved through discussion.

### 2.5 Extraction of features

**Figure 2:**
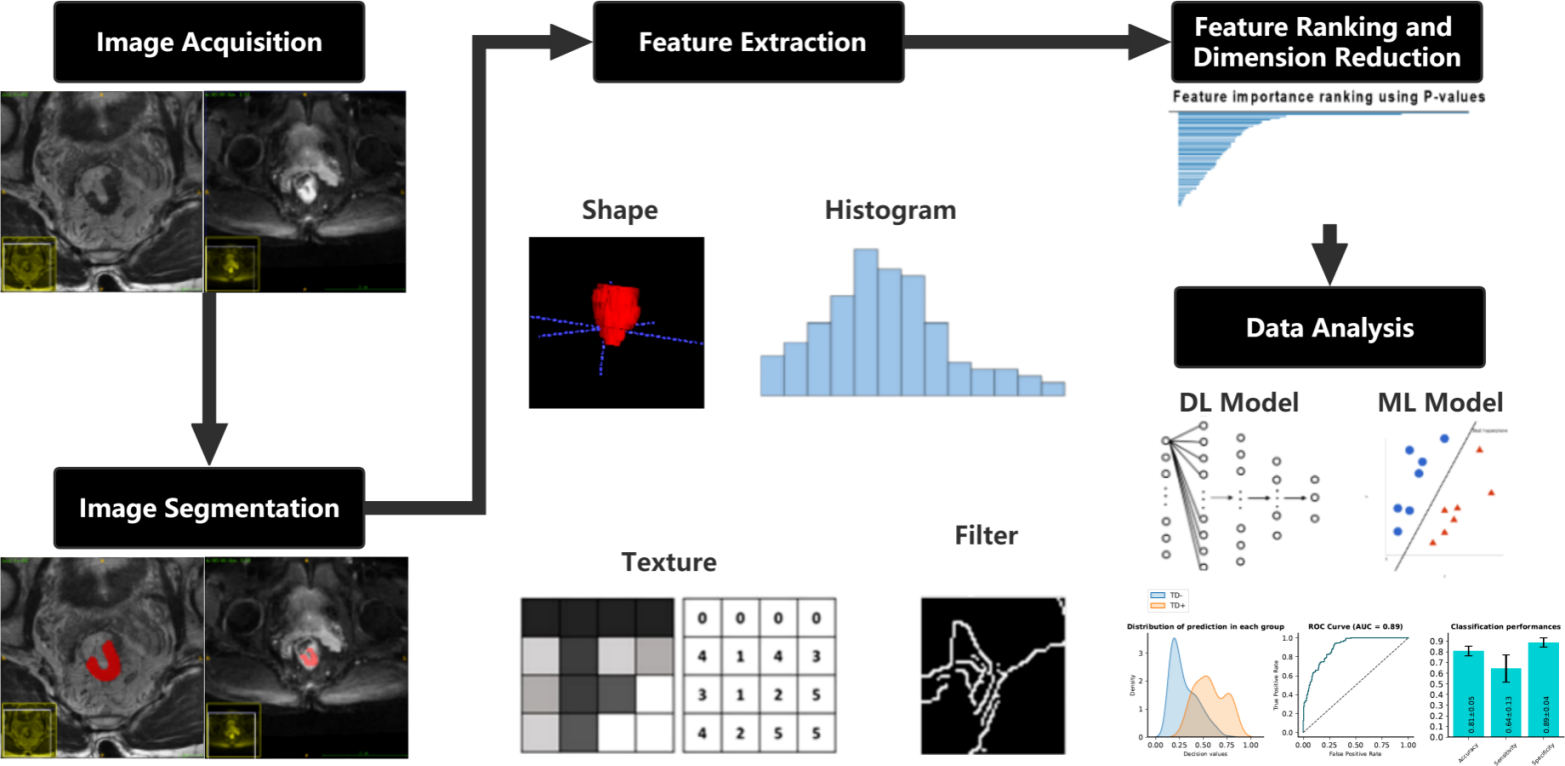
Flowchart describing the methods used in developing the clinical-DL radiomics model for TD prediction in patients with RC. DL, deep learning; TD, tumor deposition; RC, rectal cancer.

International Biomarker Standardization Initiative (IBSI)-compliant radiomic features were extracted separately for the HRT2 images and DWI images using PyRadiomics, an open-source Python package (version 2.1.2, https://pyradiomics.readthedocs.io) (31). Before feature extraction, z-score normalization of the MRI signal intensities for both the HRT2 and DWI images was performed using PyRadiomics. Consequently, 564 features were obtained for each HRT2 and DWI image, including 13 first-order statistics, 35 shape features, 9 orientation features, and 507 texture features, such as the gray-level co-occurrence matrix, gray-level size zone matrix, gray-level run-length matrix, gray-level dependence matrix, neighborhood gray-tone difference matrix, Gabor filter, Laplacian of Gaussian filter, local binary patterns, and local phase and vascularity filters. A variance test was performed on the extracted features to remove features with a low variance (<0.01). A t-test was used to estimate the radiomic features that were significantly correlated with TDs. Features with p < 0.05 were considered significant features for model development. A detailed flowchart of this process is shown in **Figure 2**.

### 2.6 Pre-processing of features

Each radiomic feature was standardized using z-score normalization to improve the robustness of the model. Missing information on the clinical characteristics was replaced with the mean value of the corresponding feature. The number of positive and negative samples was balanced using an up-sampling method within the open-source Python package Imbalanced-learn (version 0.9.0) (32).

### 2.7 Development of radiomic models

Radiomic models based on common machine learning (ML) techniques and DL methods were developed and compared to predict TDs. Three ML models and three DL models were constructed using features from HRT2 images (HRT2-ML and HRT2-DL models), DWI images (DWI-ML and DWI-DL models), and joint HRT2-DWI images (Merged-ML and Merged-DL models).

Integrated models combining radiomic information and clinical characteristics were developed to further improve the predictive performance. Three ML models and three DL models were constructed using clinical characteristics and features from HRT2 images (Clinical-HRT2-ML and Clinical-HRT2-DL models), DWI images (Clinical-DWI-ML and Clinical-DWI-DL models), and joint HRT2-DWI images (Clinical-Merged-ML and Clinical-Merged-DL models). For comparison, a clinical model that analyzed only clinical characteristics was also developed.

ML models used the least absolute shrinkage and selection operator (LASSO) technique (33) to select the optimized subset of features from 221 preprocessed features, followed by a support vector machine (SVM) to construct a prediction model.

DL models used a four-layer multi-layer perceptron (MLP) model, in which 221 preprocessed features were directly input. The feature numbers at each layer were 256, 128, 64, and 2, respectively, and the softmax activation function was used for the final output. The network model was actualized using the open-source deep learning framework PyTorch (34), where the batch size was set to 16, the learning rate was 0.001, and the Adam algorithm was used as the optimizer.

The predictive performance of each model was evaluated using five-fold repeated cross-validation. Each cross-validated split of the data was used to perform feature selection techniques to avoid bias in the estimation of the predictive performance.

### 2.8 Statistical analyses

Statistical analysis was performed with SPSS software (version 26.0; IBM, Armonk, NY) and R (version 3.5.1; R Foundation, Vienna, Austria). Differences in categorical characteristics between RC patients with and without TDs were compared using Pearson’s chi-squared test and Fisher’s exact test. Continuous variables are expressed as means ± standard deviations. Differences in continuous characteristics between the two groups were compared using the Mann-Whitney U test. For all statistical analyses, P < 0.05 (two-sided test) was considered statistically significant. The predictive performance of the models was evaluated using the area under the receiver operating characteristic (ROC) curve (AUC), over five-fold cross-validation.

## 3 Results

### 3.1 Clinical characteristics of patients

The final 500 patients included 315 males and 185 females (mean age, 64.59 ± 10.7 years). According to the pathologically confirmed TD results, the patients were divided into TD+ (n = 133) and TD− (n = 367) subgroups. The baseline characteristics of the patients are summarized in **Table 2**.

**Table 2:** The baseline characteristics of the patients.

Characteristics		TDs-positive	TDs-negative	P
Gender	Male	49(36.8)	136(37.1)	0.96
	Female	84(63.0)	231(62.9)	
Age		65.0(11.0)	64.4(10.5)	0.60
BMI		23.2(2.6)	22.9(3.2)	0.49
CEA (ng/ml)		16.5(40.3)	6.3(11.4)	<0.01
CA 19-9 (IU/ml)		61.7(300.6)	27.1(217.9)	0.16
Distance(cm)		8.8(7.2)	8.9(3.4)	0.81
Tumor length (cm)		4.6(1.4)	4.1(1.3)	<0.01
mrT stage	T1	1(0.8)	8(2.2)	<0.01
	T2	14(10.5)	101(27.5)	
	T3	86(64.7)	228(62.1)	
	T4	32(24.1)	30(8.2)	
mrN stage	N0	2(1.5)	226(61.6)	<0.01
	N1	85(63.9)	101(27.5)	
	N2	46(34.6)	40(10.9)	
CRM	Presence	44(33.1)	53(14.4)	<0.01
	Absence	89(66.9)	314(85.6)	
EVMI	Presence	57(42.9)	68(18.5)	<0.01
	Absence	76(57.1)	299(81.5)	
pT stage	T1	1(0.8)	32(8.7)	<0.01
	T2	13(9.8)	121(33.0)	
	T3	109(81.9)	184(50.1)	
	T4	10(7.5)	30(8.2)	
pN stage	N0	0(0)	272(74.1)	<0.01
	N1a	32(24.1)	38(10.4)	
	N1b	26(19.5)	35(9.5)	
	N1c	35(26.3)	0(0)	
	N2a	27(20.3)	14(3.8)	
	N2b	13(9.8)	8(2.2)	
LI	Presence	29(21.8)	19(5.2)	<0.01
	Absence	104(78.2)	348(94.8)	
PI	Presence	40(30.1)	28(7.6)	<0.01
	Absence	93(69.9)	339(92.4)	
Grade	Well differentiated	47(35.3)	181(49.3)	0.01
	Moderately differentiated	61(45.9)	145(39.5)	
	Poorly/undifferentiated	25(18.8)	41(11.2)	

Unless otherwise indicated, data are the number of patients, with percentages in parentheses. Categorical variables were compared by using the chi-squared test. P < 0.05 indicates a statistically significant difference. Continuous variables were expressed as means ± standard deviations. TD, tumor deposition; CEA, carcinoembryonic antigen; CA19-9, carbohydrate antigen 19-9; mrT stage, tumor stage on magnetic resonance imaging; mrN stage, lymph node stage on magnetic resonance imaging; CRM, circumferential resection margin; EMVI, extramural microvascular invasion; pT stage, pathological tumor stage; pN stage, pathological lymph node stage; LI, lymphovascular invasion; PI, perineural invasion; Grade, pathological tumor histological grade.

### 3.2 Performance of the radiomic models

In total, six radiomics models were developed, and the Merge-ML and DWI-DL models both demonstrated comparable performance, with AUCs of 0.69 ± 0.04 and 0.68 ± 0.03, respectively (P<0.05). The other models’ AUCs were lower than those of the aforementioned two models. The AUCs of the HRT2-ML, HRT2-DL, DWI-ML, and Merged-DL models were 0.62 ± 0.02, 0.57 ± 0.06, 0.64 ± 0.08 and 0.59 ± 0.04, respectively.

### 3.3 Performance of the integrated models combining radiomic information and clinical characteristics

**Figure 3:**
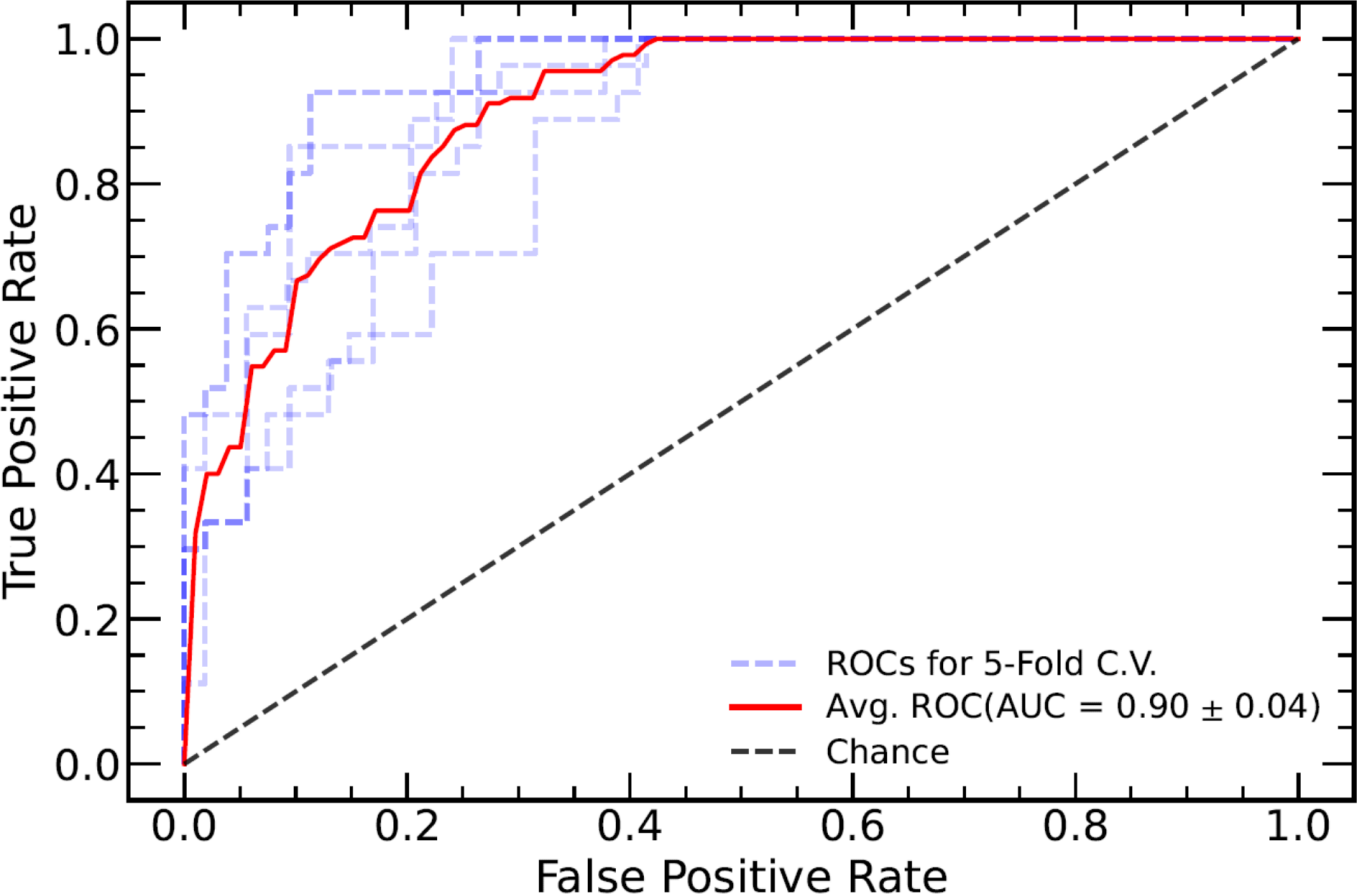
ROC curve of the combined model of DWI and clinical factors. The blue dotted lines represent the ROC curve of five-fold cross-validation (CV), and the red line represents the mean ROC curve of the five-fold CV. ROC, receiver operating characteristic; DWI, diffusion-weighted imaging.

TN staging, tumor length (measured in the sagittal view), tumor index CEA, CRM, and EMVI as assessed in the MRI report were significantly different between the TD+ and TD− groups. These clinical markers were used to establish a clinical model and were introduced into the integrated models. The performance of all models are listed in **Table 3**. Both the Clinical-ML and Clinical-DL models performed similarly, with AUC values of 0.81 ± 0.04 and 0.81 ± 0.06, respectively. Among the integrated models, the Clinical-DWI-DL model achieved the highest performance, with a diagnostic accuracy of 0.84 ± 0.05, an AUC score of 0.90 ± 0.04, sensitivity of 0.94 ± 0. 03, and specificity of 0.79 ± 0.08 (**Figure 3**). The Clinical-Merged-ML and Clinical-Merged-DL models achieved similar performances, with AUC scores of 0.83 ± 0.01 and 0.83 ± 0.05, which were both lower than the Clinical-DWI-DL model.

**Table 3:** Comparison of areas under the curve for all models.

Models	DL				ML			
	AUC	ACC	SEN	SPE	AUC	ACC	SEN	SPE
DWI	**0.68 ± 0.03**	0.674 ± 0.03	0.708 ± 0.07	0.66 ± 0.06	0.64 ± 0.08	0.64 ± 0.06	0.71 ± 0.20	0.58 ± 0.26
HRT2	0.57 ± 0.06	0.63 ± 0.05	0.57 ± 0.18	0.65 ± 0.09	**0.62 ± 0.02**	0.62 ± 0.02	0.58 ± 0.21	0.66 ± 0.22
Merged (DWI+HRT2)	0.59 ± 0.04	0.63 ± 0.07	0.66 ± 0.10	0.62 ± 0.13	**0.69 ± 0.04**	0.67 ± 0.04	0.70 ± 0.07	0.66 ± 0.06
Clinical	**0.81 ± 0.04**	0.76 ± 0.05	0.93 ± 0.05	0.72 ± 0.07	0.81 **±** 0.06	0.80 ± 0.02	0.96 ± 0.05	0.63 ± 0.07
Clinical-DWI	**0.90 ± 0.04**	0.84 ± 0.05	0.94 ± 0.03	0.79 ± 0.08	0.81 ± 0.02	0.77 ± 0.03	0.87 ± 0.04	0.68 ± 0.07
Clinical-HRT2	**0.83 ± 0.04**	0.75 ± 0.04	0.94 ± 0.04	0.69 ± 0.05	0.79 ± 0.02	0.76 ± 0.02	0.77 ± 0.06	0.75 ± 0.07
Clinical-Merged (DWI+HRT2)	**0.83 ± 0.05**	0.75 ± 0.06	0.97 ± 0.02	0.67 ± 0.08	0.83 ± 0.01	0.74 ± 0.03	0.86 ± 0.08	0.74 ± 0.07

Bold results indicate better results. The integrated model of clinical factors and diffusion-weighted imaging obtained the best performance.

### 3.4 Robustness of the model on different scanners

We compared the performance of our DWI-DL model on one scanner (GE-Signa HDxt) and all three scanners to assess the robustness of the DL-model on different scanners. We obtained AUC scores of 0.69 ± 0.08 when used with a single scanner and 0.68 ± 0.03 when used with three scanners. We did not detect a statistically significant difference (p = 0.69), even though the model performed better on a single scanner than on three scanners.

## 4 Discussion

In this study, we developed and validated a variety of models for non-invasive preoperative prediction of TDs in patients with RC, based on radiomic features, clinical factors, and a combination of both. Among all the models, the integrated DL-based model using a combination of DWI radiomic features and clinical characteristics was the most effective and achieved promising predictive performance. This approach can serve as a potential preoperative assessment tool to assist clinicians in preoperative stage evaluation and personalized treatment of patients with RC.

Of the 500 included RC patients, 26% presented with TDs, which is slightly higher than the median incidence (21.3%) of TDs in patients with CRC, as previously reported (2). TDs are an important prognostic factor in CRC, as a significantly worse prognosis has been found in patients with TDs, regardless of the sub-staging of the LNs (35). TDs are also an independent risk factor for liver, lung, and peritoneal metastases (36). Moreover, patients with TDs have a higher risk of LN metastasis and lymphovascular and perineural invasion (37). In this study, the proportion of patients with LN metastases and vascular and nerve invasion in the TD+ group (73.7%, 21.8%, and 30.1%, respectively) was also significantly higher than that in the TD− group (25.9%, 5.2%, and 7.6%, respectively), indicating a possible correlation between TDs and LN metastases, neurovascular invasion, and multi-channel tumor metastases, which are also associated with worse prognosis in patients with CRC.

While TDs cannot be reliably assessed preoperatively using traditional imaging techniques that depend on the naked eye, previous studies have shown that they may be predicted using radiomics, which provides implicit information on tumor heterogeneity far beyond the capability of visual inspection. Radiomic models based on US, CT, and MRI have been established for TD prediction. However, functional MRI (e.g., DWI) provides more information on tumor heterogeneity. Therefore, we established radiomic models based on DWI, which demonstrated higher predictive performance than HRT2-only radiomic models.

The Clinical-ML and Clinical-DL models perform similarly in **Table 3**. This is because there are only 7 clinical features, which is a relatively small number, and both DL and ML work well with such low-dimensional data. We can also observe that the Clinical-DWI-DL model improves by roughly 9% over the Clinical-DL model, while the Clinical-DWI-ML model barely improves. This could be because deep learning models outperform ML-based models in high-dimensional data situations.

A study on MRI evaluation demonstrated that a joint-modality (HRT2 and DWI) radiomic model achieved higher diagnostic performance than HRT2-only and DWI-only models (38). However, in our study, the joint-modality model did not outperform single-modality models. This result is similar to the findings of Shin et al. who predicted the complete pathological response in RC, and their joint-modality model using features from T2-weighted and DWI images had a classification performance similar to that of the T2-only model (39). We also developed an integrated model that combined radiomic features and clinical characteristics to improve the predictive performance. Among the integrated DL-based models combined with clinical factors, the model utilizing DWI-only radiomic features achieved the highest performance. This may be due to inconsistent baselines and different spatial resolutions between HRT2 and DWI scans of RC, which cannot be reduced by spatial resampling prior to feature extraction.

In addition to investigating the model’s performance, we investigated its robustness due to the complexity of clinical data collection. We chose the model with the best performance (DWI-DL) for this investigation because clinical information is independent of the scanner, allowing us to test the model’s robustness across a range of scanners. We found no significant differences in the radiomics model across machines (AUCs of the single- and multi-scanner models: 0.69 ± 0.08 and 0.68 ± 0.03, respectively, with P = 0.69), indicating good robustness of our radiomics model.

This study has some limitations. First, to our knowledge, this is the largest study to date on TD radiomics research, but it is still not large enough to avoid selection bias that compromises the generalization ability of our models. Second, this retrospective study excluded patients who had received neoadjuvant chemotherapy or radiotherapy before surgery, which introduced a further selection bias. Third, this was a single-center study, and the difference in sample sizes between the TD+ and TD− groups was large. Therefore, further prospective, multicenter studies with larger cohorts are warranted to improve prediction outcomes and define the potential standardization of our models.

## 5 Conclusions

Our integrated model combining clinical variables (tumor markers and MRI reporting status) and MRI radiomic features in a DL model can non-invasively and preoperatively predict TDs in patients with RC. In particular, the model that used DWI and clinical features showed the highest predictive performance. This model can serve as a potential preoperative assessment tool in clinical practice for more effective tumor staging and risk stratification to provide optimal treatment for patients with RC.

## Data availability statement

The original contributions presented in the study are included in the article/supplementary material. Further inquiries can be directed to the corresponding authors.

## Author contributions

CF, LZ and FZ conceived of the presented idea. MH, JQ, PL, ZY, KS, MW, XW and FL collected the data. LZ, CF, JZ, WL and TS analyzed the data. FL drafted the manuscript. TS and JS improved the quality of English. All authors contributed to the article and approved the submitted version.
